# A307 NEUROGASTROENTEROLOGY & MOTILITY CAID (CHRONIC ATRIAL AND INTESTINAL DYSRHYTHMIA) SYNDROME: A SEVERE LATE-ONSET PEDIATRIC INTESTINAL PSEUDO-OBSTRUCTION

**DOI:** 10.1093/jcag/gwad061.307

**Published:** 2024-02-14

**Authors:** l adouane, P Poinsot, J Castilloux, C Faure

**Affiliations:** Centre Hospitalier Universitaire Sainte-Justine, Montreal, QC, Canada; Centre Hospitalier Universitaire Sainte-Justine, Montreal, QC, Canada; Centre Hospitalier de l'Universite Laval, Quebec, QC, Canada; Centre Hospitalier Universitaire Sainte-Justine, Montreal, QC, Canada

## Abstract

**Background:**

Pediatric intestinal pseudo-obstruction (PIPO) is a rare, heterogeneous and severe gut motility disorder. It is associated with a high morbidity and mortality rate. The etiology of PIPO can be either primary (sporadic or familial), secondary or idiopathic. In primary forms, 50 to 75% of patients will present symptoms in the first month of life and 80% by the end of the first year of life. In 2014, Chetaille et al. described the CAID syndrome related to a recessive SGOL1 mutation as a new form of PIPO associated with cardiac dysrhythmia.

**Aims:**

Our aim is to describe the clinical presentation and the specific digestive and nutritional features of pediatric patients with SGOL1 mutation.

**Methods:**

Monocentric, retrospective and descriptive study. We enrolled children and adolescents aged less than 18 years. All data were collected from patients’ files.

**Results:**

8 patients were included (5 girls), two of them were first-degree related. They all had a typical clinical presentation of PIPO (excessive abdominal distension, abdominal pain and vomiting) but with a later onset than usually seen in primary PIPO (median (range) age: 6.3 years [2-15.6]). The median age (range) at diagnosis was 7.8 years [4-15.7]. In most patients contrast studies showed massively distended small bowel and colon. Antroduodenal manometry was performed in 6 patients and was abnormal with a neuropathic pattern. Others were diagnosed using the ESPGHAN criteria (2018 Jun;66(6):991-1019). All patients needed parenteral nutrition at a median (range) age of 11.8 years [6.5-15.9] due to failure to thrive, intestinal failure and electrolytic disorders. Seven patients needed an ileostomy. One out of 8 patients was off PN at 18 years. Three patients had sinus dysfunction: one at the time of PIPO diagnosis and two sometimes after. All of them required a pacemaker. One patient died in her twenties and it wasn’t related to PIPO or its possible complications.

**Conclusions:**

Recessive SGOL1 mutation, a new cohesinopathy affecting the gut and heart rhythm, represents a late-onset but severe form of PIPO. It is usually associated with sinus dysfunction and cardiac dysrhythmia. However, digestive symptoms appear first. Long term outcome is still to describe.

**Funding Agencies:**

None

